# Chalcomoracin prevents vitreous‐induced activation of AKT and migration of retinal pigment epithelial cells

**DOI:** 10.1111/jcmm.16590

**Published:** 2021-08-25

**Authors:** Haote Han, Yanhui Yang, Bing Liu, Jingkui Tian, Lijun Dong, Hui Qi, Wei Zhu, Jiantao Wang, Hetian Lei

**Affiliations:** ^1^ Institute of Cancer and Basic Medicine Chinese Academy of Sciences Zhejiang Cancer Hospital Cancer Hospital of the University of Chinese Academy of Sciences Hangzhou China; ^2^ College of Biomedical Engineering & Instrument Science Zhejiang University Hangzhou China; ^3^ Schepens Eye Research Institute of Massachusetts Eye and Ear Boston MA USA; ^4^ Department of Ophthalmology Harvard Medical School Boston MA USA; ^5^ School of Basic Medical Sciences Ningxia Medical University Yinchuan China; ^6^ Guangzhou Women and Children's Medical Center Guangzhou Medical University Guangzhou China; ^7^ Shenzhen Eye Hospital Shenzhen Eye Institute Jinan University Shenzhen China

**Keywords:** Akt, chalocomoracin, contraction, migration, p53, proliferation, proliferative vitreoretinopathy, vitreous

## Abstract

Retinal pigment epithelial (RPE) cells are the major cell type in the epi‐ or sub‐retinal membranes in the pathogenesis of proliferative vitreoretinopathy (PVR), which is a blinding fibrotic eye disease and still short of effective medicine. The purpose of this study is to demonstrate whether Chalocomoracin (CMR), a novel purified compound from fungus‐infected mulberry leaves, is able to inhibit vitreous‐induced signalling events and cellular responses intrinsic to PVR. Our studies have revealed that the CMR IC50 for ARPE‐19 cells is 35.5 μmol/L at 72 hours, and that 5 μmol/L CMR inhibits vitreous‐induced Akt activation and p53 suppression; in addition, we have discovered that this chemical effectively blocks vitreous‐stimulated proliferation, migration and contraction of ARPE‐19 cells, suggesting that CMR is a promising PVR prophylactic.

## 1 INTRODUCTION

Open eye trauma is a type of mechanical eye trauma (the other type is closed eye trauma), which is caused by the penetration of foreign sharp instrument or blunt force to make the full thickness of the eyeball wall open.^1^ Rhegmatogenous retinal detachment (RRD), an open eye trauma, is an important cause of blindness, occurring due to a full‐thickness break in the neurosensory retina.^2^ In the United States, approximately 55 000 people suffer from RRD,^3^, ^4^ and there are approximately 203 000 open eye injuries worldwide each year.^5^ Proliferative vitreoretinopathy (PVR) is the most common cause of recurrent retinal detachment after retinal detachment repair, following RRD and occurs in 5%‐11% of patients.^6^ PVR is still the main reason for failure after RRD surgery, and since the initial description, no relevant progress has been made in clinical management.^7^ In addition, so far, there is no effective drug to prevent the formation of PVR.^8^, ^9^, ^10^ Therefore, pharmacological approaches for PVR treatment are urgently demanding.

Proliferative vitreoretinopathy was characterized by retinal detachment and membrane growth.^3^ Retinal pigment epithelial cells (RPEs) are considered to be important and central roles in the pathogenesis of PVR. When the retina is detached by contraction,^11^ retinal cells (eg RPEs) form the epi‐ or sub‐ retinal membranes undergoing a series of responses including proliferation and migration.^12^ Hypothesis has been made that an eclectic range of chemical factors present within the vitreous fluid could be mitogenic to RPEs.^13^ This hypothesis was supported by the observation showing that vitreous aspirated from patients with PVR was able to stimulate the migration of RPEs.^14^ Moreover, vitreous fluid was shown to have an effect on cell proliferation which appeared to be mediated through TGF‐β.^15^ Therefore, the vitreous‐treated RPEs can be applied to in vitro studies of PVR.

Phosphoinositide 3 kinases (PI3Ks) play a critical role in experimental PVR^16^ and regulate AKT, which is an oncogene product known as protein kinase B.^17^, ^18^, ^19^ Activation of AKT can initiate multiple cellular processes such as cell survival, proliferation, growth and migration.^20^, ^21^ More recently, specific genetic variants located at p53 gene have been identified as significant risk factors for PVR development in patients.^22^, ^23^ Vitreous from experimental rabbits (RV) or vitreous from patients with PVR activate the signalling pathway of phosphoinsositide 3 kinase (PI3K)/Akt, which phosphorylates Mdm2, resulting in p53 degradation.^16^, ^24^ Prevention of Mdm2 interaction with p53 suppresses experimental PVR.^25^

**FIGURE 1:**
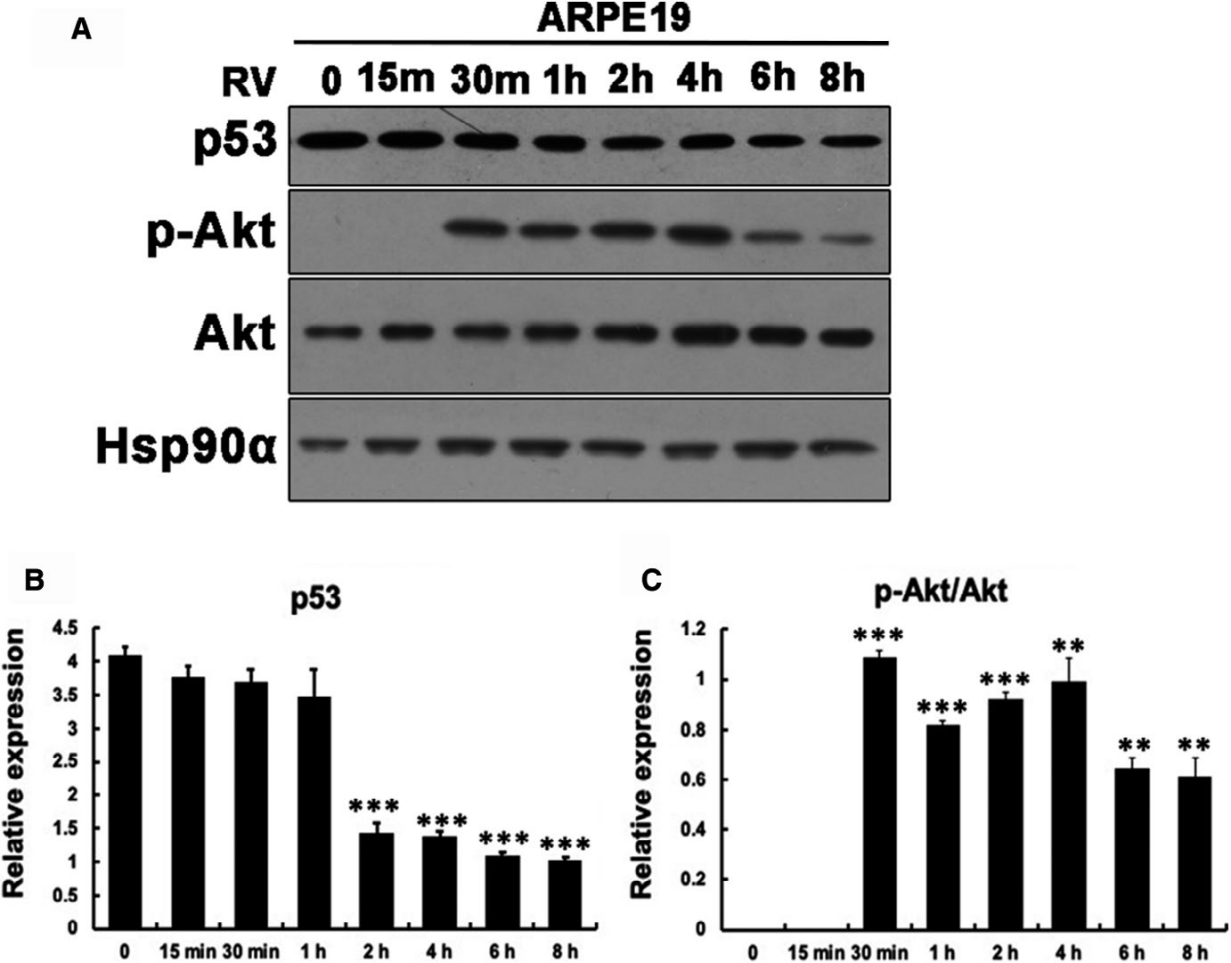
Vitreous induces activation of AKT and reduction of p53 in ARPE‐19. A, Serum‐starved ARPE‐19 cells were treated with RV for 15 min, 2 h, 4 h, 6 h, 8 h, 10 h, 12 h and 24 h. Their lysates were subjected to Western blotting analysis using indicated antibodies. RV: experimental‐rabbit PVR vitreous diluted 1:3 in DMEM/F12. B, C, Quantitation of the intensity of bands resulted from Western blot. The intensity of the p53, p‐Akt and Akt bands in (A) was first normalized to that of the corresponding Hsp90α bands and then the ratio of p‐Akt/Akt were calculated. The bar graphs are mean ± SD of three independent experiments. The data were analysed using one‐way ANOVA followed by the Tukey HSD post hoc test. **P < .01 vs 0, ***P < .001 vs 0

Chalocomoracin (CMR; MW: 648.69; structure, Figure 1C) is a major secondary metabolite produced by fungus‐infected mulberry leaves that protects the leaves by suppressing fungal germination.^26^ Recently, CMR has been reported to have a broad spectrum of biological activities against rhinovirus, methicillin‐resistant Staphylococcus aureus (MRSA)^27^, ^28^, ^29^ and human cancer cell lines,^30^, ^31^ but there has not yet been any thorough investigation into its effects on PVR. Herein, we show that CMR inhibits vitreous‐stimulated activation of Akt and abrogates vitreous‐induced p53 suppression, proliferation, migration and contraction of ARPE‐19, suggesting CMR is a promising medicine for prevention of PVR.

## 2 MATERIALS AND METHODS

### 2.1 Major reagents

As determined by HPLC/UV‐visible, 98.5% pure Chalocomoracin (CMR) was isolated by Jingkui Tian's laboratory, the Key Laboratory of Biomedical Engineering, Zhejiang University. The compounds were dissolved in pure DMSO and stored at −20°C prior to use. Primary antibodies against p‐Akt, Akt and p53 were purchased from Cell Signaling Technology, and β‐Actin was ordered from Santa Cruz Biotechnology. Horseradish peroxidase‐conjugated mouse anti‐rabbit IgG, and goat anti‐mouse IgG were ordered from Santa Cruz Biotechnology. Enhanced chemiluminescent substrate to detect horseradish peroxidase was purchased from Thermo Scientific.

ARPE‐19 cells (American Type Culture Collection) was cultured in Dulbecco's modified Eagle's medium/nutrient mixture (DMEM/F‐12, Invitrogen) supplemented with 10% foetal bovine serum (FBS). Cells were cultured in a humidified incubator at 37°C and 5% CO_2_.

Detailed protocols of Western blot, cell proliferation assay, cell migration assay and collagen contraction assay were descripted in our previous report in.^32^

### 2.2 Preparation of rabbit vitreous

Rabbit vitreous was prepared from frozen rabbit eyeballs as described previously.^33^ In short, the vitreous was cut out from the eyeball while it was still frozen, transformed into melting and then centrifuged at 13 000 *g* at 4°C for 2 minutes. The resulting supernatant was used.

### 2.3 Western blot

Briefly, when cultures reached 90% confluence in 24‐well plates, they were serum‐starved overnight followed by treatment with vitreous (0‐24 hours) from rabbits with PVR (RV, diluted 1:3 in DMEM/F12) or with CMR (5 μmol/L) for 8 hours. After being rinsed twice with ice‐cold phosphate‐buffered saline (PBS), cell lysates were harvested by sample buffer which was diluted with extraction buffer (10 mmol/L Tris‐HCl, pH 7.4, 5 mmol/L EDTA, 50 mmol/L NaCl, 50 mmol/L NaF, 1% Triton X‐100, 20 μg/mL aprotinin, 2 mmol/L Na3VO4, and 1 mmol/L phenylmethylsulfonyl fluoride) from 5 × protein sample buffer [(25 mmol/L EDTA (pH 7.0), 10% sodium dodecyl sulphate (SDS) (Sigma‐Aldrich Corp.), 500 mmol/L dithiothreitol, 50% sucrose, 500 mmol/L TrisHCl (pH 6.8) and 0.5% bromophenol blue)]. Then, we boiled samples for 5 minutes and centrifuged them for 5 minutes at 13 000 *g*. Subsequently, proteins in samples were separated by 10% SDS‐PAGE, transferred to polyvinylidene difluoride membranes and subjected to Western blot analyses. Experiments were repeated at least three times. Signal intensity was determined by densitometry with ImageJ software.^33^

### 2.4 Cell viability assay

Cell viability was assessed by the 3‐(4,5‐dimethylthiazol‐2‐yl)‐2,5‐diphenyl‐2H‐tetrazolium bromide (MTT, Solarbio, M8180). ARPE‐19 cells were seeded into 96‐well plates containing their respective medium supplemented with 10% FBS and incubated for 24 hours. The cells were then exposed to a range of concentrations of CMR (0, 5, 10, 20 and 40 μmol/L) dissolved in DMSO in 5% FBS‐supplemented medium for 24‐72 hours. Following this, the medium was removed and replaced with 200 μL of 0.5 mg/mL MTT in 10% FBS‐containing medium, and the cells were incubated in the 5% CO_2_ incubator at 37°C for 1 hour. Supernatants were removed from the wells, and the MTT dye was solubilized in 200 μL/well DMSO (Sigma, 67‐68‐5). Absorbance was measured at 570 nm on a plate reader. Each condition was performed with 6 replicates, and all assays were performed in triplicate, and the results of assays were presented as mean ± SD.

### 2.5 Phase contrast imaging

ARPE‐19 cells were seeded into a 24‐well plate containing their respective medium supplemented with 10% FBS and incubated for 24 hours. The cells were then exposed to a range of concentrations of CMR (0, 5, 10, 20 and 40 μmol/L) dissolved in DMSO in 5% FBS‐supplemented medium for 24‐72 hours. The morphology of ARPE‐19 was detected by EVOS Cell Imaging System.

### 2.6 Cell proliferation assay

ARPE‐19 were plated into 24‐well plates at a density of 3 × 10^4^ cells/well in DMEM/F12 with 10% FBS. After attachment, cells were treated with RV (1:3 dilution in DMEM/F12) in addition to CMR (5 μmol/L). 24 hours later, ARPE‐19 were digested and counted in a light microscope. This experiment was conducted at least three times.

### 2.7 Cell migration assay

Briefly, when cells grew to confluence in 24‐well plates, autoclaved 200 μL pipet tips were used to scratch the cells across the wells. Cells were treated with RV (1:3 dilution in DMEM/F12) plus or minus CMR (5 μmol/L) after rinsing with PBS. The scratched areas were photographed at 0 and 24 hours. As mentioned earlier,^32^ the data were analysed using Image J and Adobe Photoshop CS4 software. Three independent experiments were conducted at least.

### 2.8 Collagen contraction assay

ARPE‐19 were re‐suspended in 1.5 mg/mL of neutralized PureCol type I bovine collagen solution (Advanced BioMatrix) (pH 7.2) on ice at a density of 1 × 10^6^. Then, the cells in collagen solution were transferred into 5 mg/mL bovine serum albumin (BSA)/PBS pre‐incubated 24‐well plates and incubated at 37°C for 90 minutes for polymerization. Subsequently, 0.5 mL DMEM/F12 or RV (1:3 dilution in DMEM/F12) with or without CMR (5 μmol/L) was added into the plates. Take photographs of the plates on day 3. The gel area was calculated using a formula 3.14 × r^2^, where r is the radius of the gel.

### 2.9 Statistics

Data were analysed as described preciously.^32^ At least three independent experiments were analysed using ordinary one‐way ANOVA followed by the Tukey honest significant difference (HSD) post hoc test. A *P* value less than 0.05 was considered significantly difference.

## 3 RESULTS

### 3.1 Vitreous induces activation of AKT and reduction of p53 in ARPE‐19

Based on the previous reports, vitreous can promote the formation of PVR.^14^ We used vitreous from PVR rabbits to establish a model in vitro. ARPE‐19 were seeded into the 24‐well plates, and they were serum‐starved overnight followed by treatment with vitreous (0‐24 hours) from rabbits with PVR (RV, diluted 1:3 in DMEM/F12). Then, cells were collected and performed Western blot. In terms of the activation of AKT, we found that RV (1:3 dilution in DMEM/F12) can significantly promote the phosphorylation of AKT at 30 minutes, and this process can be maintained for 8 hours, but the highest level achieves at 4 hours (Figure 1A, B). We mentioned earlier period during the formation of PVR, the expression of p53 was suppressed. Our results indicated that, in ARPE‐19 treated with RV, obvious inhibition effect of p53 was observed after 1 hour followed by the lowest level at 8 hours. Therefore, we chose 8 hours‐treatment of RV in ARPE‐19 for the following experiments.

### 3.2 CMR prevents vitreous‐induced activation of AKT and reduction of p53

**FIGURE 2:**
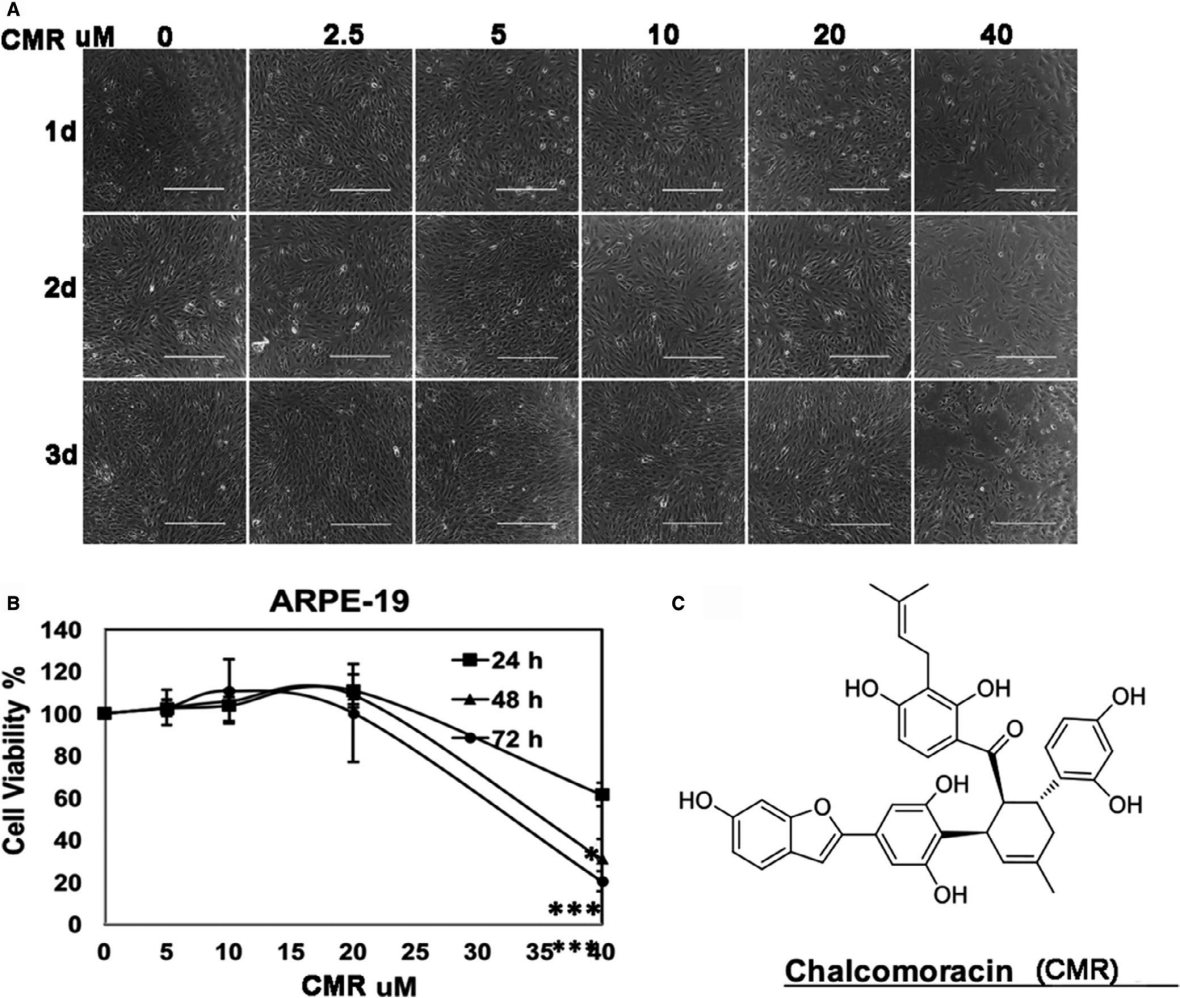
Evaluation of CMR toxicity to ARPE‐19 cells. A, Confluent ARPE‐19 in a 24‐well plate were treated with a serially increasing concentration of CMR (V, 2.5, 5, 10, 20 and 40 μmol/L) in 5% FBS DMEM/F12. The images shown were taken of ARPE‐19 treated for 24 h, 48 h and 72 h. V: vehicle 0.1%DMSO. Scale bar: 200 μm. This is representative of three independent experiments. B, Dose‐dependent inhibition on the viability of ARPE‐19 was detected by MTT assay, following treatment for 24‐72 h in 5% FBS‐supplemented medium. Points: mean; bar: SD. Data are expressed as percentages of viable cells (treated vs control). All assays were performed in triplicate. The data were analysed using one‐way ANOVA followed by the Tukey HSD post hoc test. **P < .01 vs 0, ***P < .001 vs 0. C, CMR structure

To find the potential effect of CMR for preventing PVR pathogenesis, we investigated the cytotoxiciity of CMR to ARPE‐19. Thus, we treated cells with serially increasing concentrations: 5, 10, 20 and 40 μmol/L. The results showed that 40 μmol/L CMR became obviously toxic to ARPE‐19 cells indicated by morphology (eg cells shrinking and cell numbers) (Figure 2A). In these cells, MTT assay (Figure 2B) demonstrated that the IC_50_ of CMR was 35.5 μmol/L at 72 hours. However, 5 μmol/L CMR did not show obvious toxicity to ARPE‐19 cells, which was assessed by morphology and MTT assay (Figure 2).

**FIGURE 3:**
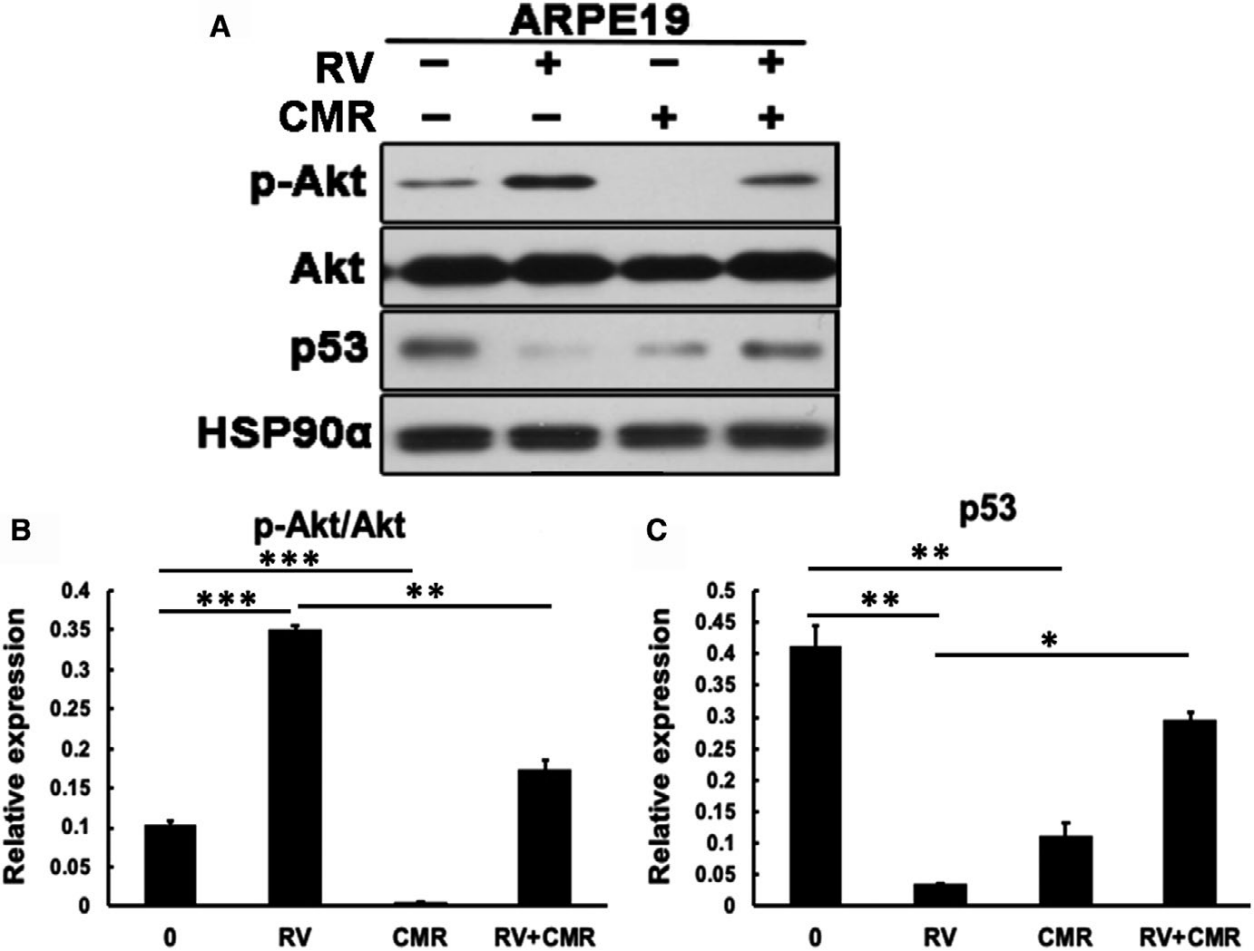
CMR prevents vitreous‐induced activation of AKT and reduction of p53. A, Serum‐starved ARPE‐19 cells were treated with vitreous in addition to CMR (5 μmol/L) for 8 h. RV: experimental‐rabbit PVR vitreous diluted 1:3 in DMEM/F12. Their lysates were subjected to Western blotting analysis using indicated antibodies. This is representative of at least three independent experiments. B, C, Quantitation of the intensity of bands resulted from Western blot. The intensity of the p53, p‐Akt and Akt bands in (A) was first normalized to that of the corresponding Hsp90α bands and then the ratio of p‐Akt/Akt were calculated. The bar graphs are mean ± SD of three independent experiments. The data were analysed using one‐way ANOVA followed by the Tukey HSD post hoc test. *P < .05 vs 0, **P < .01 vs 0, ***P < .001 vs 0

To examine the impacts of CMR on the AKT/p53 pathway, we monitored phosphorylation of AKT at serine 473 as an indirect measure of activation of AKT. Based on previous study (Figures 1, 2), we treated ARPE‐19 cells with CMR (5 μmol/L) in addition to RV for 8 hours. Western blot analysis of their lysates showed that CMR at 5 μmol/L significantly blocked vitreous‐induced activation of AKT (Figure 3A, B) and suppressed vitreous‐inhibited impact on p53 expression.

### 3.3 CMR prevents vitreous‐induced proliferation of ARPE‐19

**FIGURE 4:**
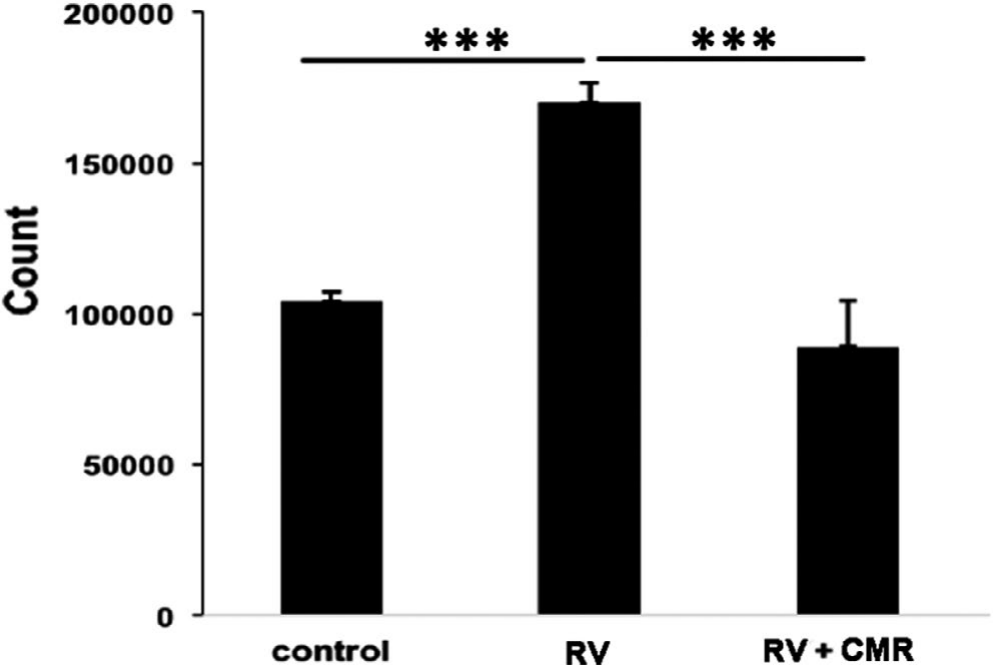
CMR diminishes vitreous‐induced cell proliferation. ARPE‐19 cells were seeded into a 24‐well plate at a density of 3 × 10^4^ cells/well in DMEM/F12 + 10% FBS. When attaching the plate, the cells were treated with 0.5 mL DMEM (−) or RV supplemented with CMR (5 μmol/L). After treatment for 48 h, the cells were counted with a haemocytometer under a light microscope. The mean ± SD of the three independent experiments is shown. *** denotes *P* < .001

PI3K/Akt signalling transduction can trigger cellular responses such as cell proliferation. In addition, another study has proved that CMR can inhibit the proliferation of breast cancer cells MDA‐MB‐231(Figure S1). Thus, we examined whether CMR could block vitreous‐mediated cell proliferation, a characteristic of the pathogenesis of PVR.^25^ As shown in Figure 4, RV stimulated (1.6 ± 0.2 fold) proliferation of ARPE‐19, and CMR completely abrogated the RV‐mediated cell proliferation.

### 3.4 CMR blocks vitreous‐induced migration of ARPE‐19

**FIGURE 5:**
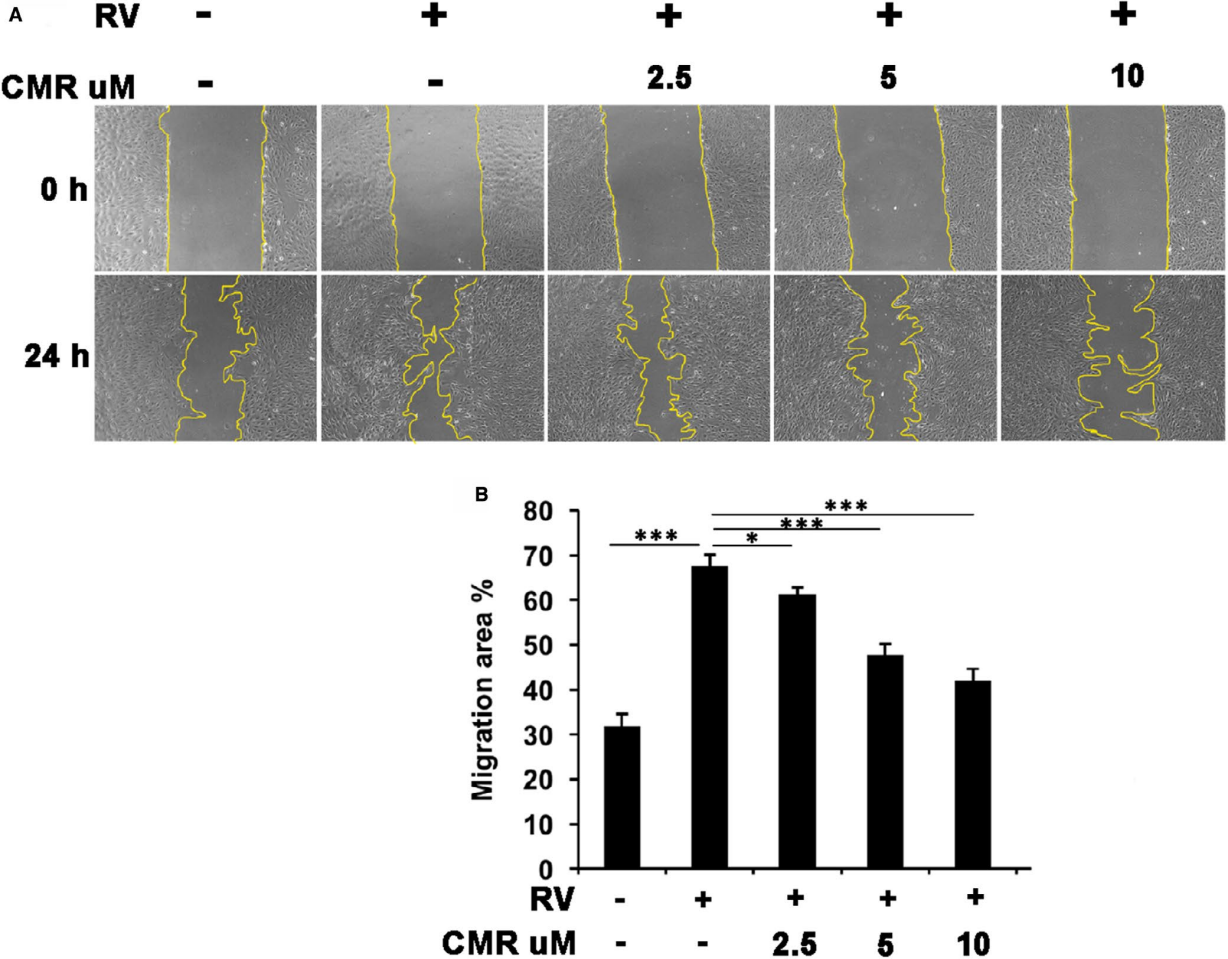
CMR abrogates vitreous‐induced cell migration. When cells grew to confluence in 24‐well plates, autoclaved 200 μL pipet tips were used to scratch the cells across the wells. Cells were treated with RV (1:3 dilution in DMEM/F12) plus or minus CMR (5 μmol/L) after rinsing with PBS. The scratched areas were photographed at 0 and 24 h and analysed for the scratched areas using image J and Adobe Photoshop CS4 software. The data of bar graphs are the mean ± SD of three independent experiments. A, The raw pictures of three independent experiments are shown (B) * denotes *P* < .05, *** denotes *P* < .001 using one‐way ANOVA followed by the Tukey HSD post hoc test

In PVR, RPEs have better migration and proliferation ability under the stimulation of growth factors. We found that CMR has a good effect on inhibiting the migration and invasion of MDA‐MB‐231 cells at the concentration of 2 μmol/L and 4 μmol/L (Figure S2, S3). Therefore, we next investigated whether CMR could inhibit RV‐induced cell migration. As shown in Figure 5, RV promoted migration of ARPE‐19 in a wound‐healing assay (Figure 5A), and CMR significantly prevented this RV‐induced cellular event through a dose‐dependent way (Figure 5B).

### 3.5 CMR blocks vitreous‐induced contraction of ARPE‐19

**FIGURE 6:**
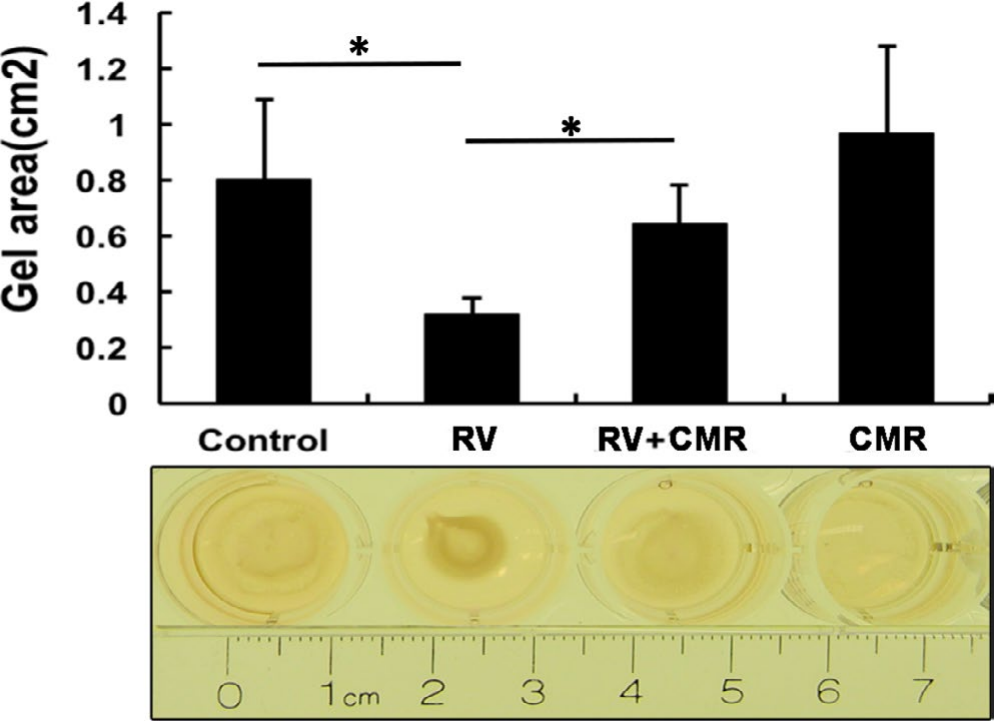
CMR blocks RV‐stimulated contraction of ARPE‐19. The ARPE‐19 cells were re‐suspended in 1.5 mg/mL of neutralized collagen I (pH 7.2) at a density of 1 × 10^6^ cells/mL and seeded into wells of a 24‐well plate that had been pre‐incubated overnight with 5 mg/mL (BSA/PBS). The collagen was solidified by incubation at 37°C for 90 min. The polymerized gels were overlaid with DMEM/F12 alone (−) or RV supplemented with CMR (5 μmol/L) or its vehicle as indicated. 48 h later, the gel diameter was measured and the gel area calculated using the formula: 3.14 × (diameter/2)2. The bar graphs represent the mean ± SD of the three independent experiments; *denotes *P* < .05 using one‐way ANOVA followed by the Tukey HSD post hoc test. A photograph of the representative experiment in A is shown at the bottom of the bar graphs

In the pathogenesis of PVR, another characteristic of PVR is contraction which causes a retinal re‐detachment.^11^ So, we attempted to apply the collagen gel contraction test to simulate this clinical event to find drugs that may prevent the pathogenesis of PVR. As predicted, CMR completely blocked the vitreous‐induced ARPE‐19 contraction (Figure 6A, B). Taken together, our findings indicate that CMR is a potent PVR prophylactic.

## 4 DISCUSSION

Our results herein show that RV triggers the pathway of Akt/P53 in ARPE‐19 cells, and that this pathway is critical for RV‐stimulated cellular responses (eg proliferation, migration and contraction). More importantly, these experiments demonstrate that CMR inhibits vitreous‐induced Akt activation and p53 suppression in ARPE‐19 cells, and prevents vitreous‐stimulated cell proliferation, migration and contraction of ARPE‐19 cells in the pathogenesis of PVR. In this study, we used ARPE‐19, which is a spontaneously arising RPE cell line derived from the normal eyes of a 19‐year‐old male donor, because these cells still express RPE specific markers including cellular retinaldehyde‐binding protein (CRALBP) and RPE 65 kD protein (RPE65).^32^, ^34^, ^35^

The pathogenesis of PVR is divided into several stages: (a) migration of cells, mainly RPE and glial cells; (b) proliferation of the migrating cells; (c) membrane development; (d) contraction of the cellular membrane; (e) extracellular collagen production; and (f) creation of fixed folds in the retina.^36^ Based on the idea that cellular proliferation is the main feature of PVR, many researchers have tried to solve this problem by inhibiting the proliferation of cells, but this problem remains unsolved. So, we can now focus on other mechanisms involved in PVR with the aim of developing a pharmacological approach for PVR treatment.

**FIGURE 7:**
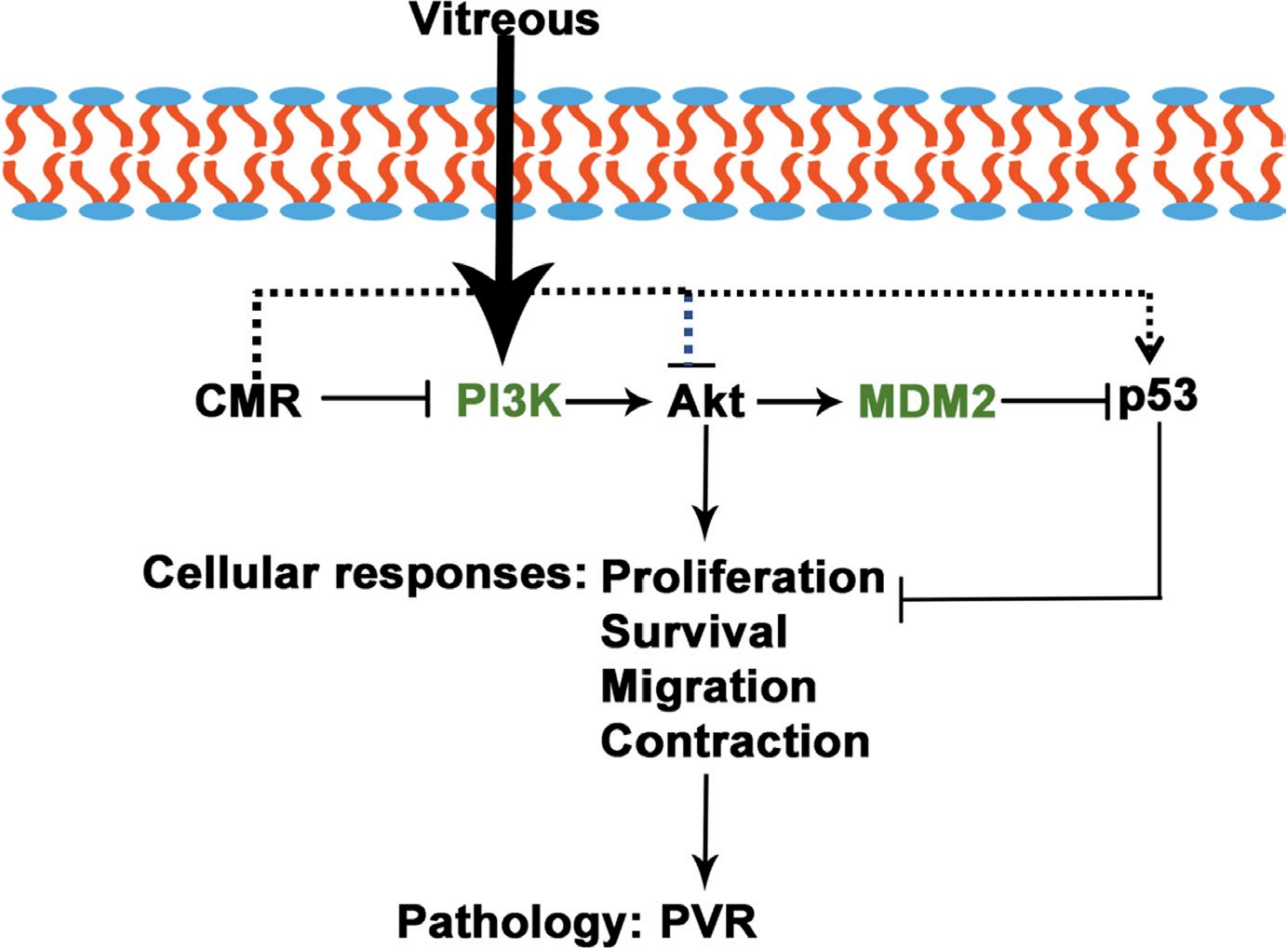
Diagram of CMR inhibiting vitreous‐induced activation of Akt/p53 signalling pathway and PVR‐related cellular responses including proliferation, migration and contraction

In our previous study, antioxidants were found to prevent intracellular signalling events that are essential for experimental PVR. For example, N‐acetyl‐cysteine (NAC) was shown to be capable of protecting rabbits from PVR^37^ and prevented the vitreous‐driven PDGFRα activation and the inherent cellular response of PVR (collagen gel contraction and cell proliferation),^37^ but the effect dose of NAC is too high (10 mmol/L). Another report showed that idelalisib, a specific inhibitor of PI3Kδ, specifically inhibited vitreous‐induced Akt activation in RPE cells from epiretinal membranes from patients with PVR (RPEMs), as well as prevented vitreous‐stimulated cell proliferation, migration and contraction of RPEMs.^38^ Idelalisib at 1 μmol/L completely inhibited vitreous‐induced activation of Akt, but it did not block Erk activation at its 10 μmol/L concentration,^38^ suggesting that idelalisib specifically blocked vitreous‐stimulated activation of Akt.^38^, ^39^ Given that the formation of PVR is a complicated process, here we revealed for the first time that, as a natural compound, CMR has the advantage of effective, multi‐target and low toxicity, in terms of blocking vitreous‐induced cellular responses contributes to PVR. CMR has been shown as a broad spectrum of biological activities, but there has not yet been any thorough investigation into the molecular mechanisms of CMR involved in different diseases except human reproductive system cancer. In our previous study, CMR induced paraptosis in combination with the mitophagy pathway in prostate and breast cancer cell lines without influencing the normal cells.^31^ Then, we found that CMR could inhibit cell proliferation, invasion and migration of MDA‐MB‐231 cells (Figure S1, S2, S3). Therefore, we hypothesize that CMR can inhibit PVR, which is closely related to proliferation and migration of RPEs. As predicted, in the current study, we found an inhibitory effect of CMR on Akt activation and p53 inactivation that was stimulated by vitreous in our study model (Figure 7) and that CMR repressed the vitreous‐induced proliferation, migration and contraction of ARPE‐19 cells (Figures 4, 5, 6). These data demonstrate that CMR prevents the PVR pathogenesis focussing on inhibiting activation of AKT and suppression of p53, which is main molecular mechanism detected in PVR.^20^, ^21^, ^23^ In Figure 3, CMR treatment alone can reduce p‐Akt and softly inhibit the expression of p53. Based on the in fact, CMR itself has the effect of inhibiting cancer cell proliferation, and the regulation may be a multi‐target pathway. Therefore, CMR treatment alone has an impact on Akt signalling pathway. In this study, we examined the treatment of vitreous with CMR, which simulated an intervention process in the incomplete or early stage of PVR, so CMR can be used as a preventive treatment for PVR. Other treatment methods of CMR need to be studied in the future.

PDGF‐BB has been reported to be capable to induce the underlying pathways of Erk, p38, JNK activation in PVR,^40^ on the other hand, TGFβ induces epithelial mesenchymal transition (EMT)^41^, ^42^ in RPEs. Thus, many signalling pathways are activated in PVR pathogenesis, of which especially Akt/MDM2/p53 among those signals is hyperactive in the vitreous environment.^32^, ^39^

## 5 CONCLUSIONS

Our data were clearly evident that CMR at 5 μmol/L completely blocked vitreous‐induced activation of Akt, and p53 attenuation, however, whether it can suppress the activation of Erk, p38 or JNK induced by vitreous remains unknown. Furthermore, the impacts of CMR on vitreous‐induced EMT also need further investigation. Overall, our work indicates CMR can act as a potential PVR prophylactic, addressing a currently unmet clinical need.

## CONFLICT OF INTEREST

HH, JT and WZ have a patent with CMR, and all other authors declare that they have no conflicts of interest with the contents of this article.

## AUTHOR CONTRIBUTIONS

**Haote Han:** Data curation (lead); Formal analysis (lead); Investigation (lead); Methodology (lead). **Yanhui Yang:** Data curation (equal); Formal analysis (equal). **Bing Liu:** Data curation (equal). **Jingkui Tian:** Funding acquisition (equal). **Lijun Dong:** Investigation (equal); Methodology (equal); Validation (equal). **Hui Qi:** Investigation (equal); Methodology (equal); Validation (equal). **Wei Zhu:** Data curation (equal); Funding acquisition (equal). **Jiantao Wang:** Funding acquisition; Project administration (equal). **Hetian Lei:** Funding acquisition; Project administration (equal).

## Supporting information

Supplementary Material

## Data Availability

The data that support the findings of this study are available from the corresponding author upon reasonable request.
