# Supplementary material for: Chalcomoracin prevents vitreous‐induced activation of AKT and migration of retinal pigment epithelial cells
Source: J Cell Mol Med. 2021 Aug 25;25(19):9102–11. doi: 10.1111/jcmm.16590 (PMC8500972; doi:10.1111/jcmm.16590)
Supplement: Supplementary file 1 — Supplementary Material [file JCMM-25-9102-s001.pdf]

Chalcomoracin prevents vitreous-induced activation of AKT and migration of retinal pigment epithelial cells

Haote Han[1,2, 3, 4, #], Yanhui Yang [5, 3, 4#], , Bing Liu[4, 5, 7], Jingkui Tian[1, 2], Lijun Dong[7], Hui Qi[7], Wei Zhu[1, 2*], Jiantao Wang[6*] and Hetian Lei[6,3,4*]

[1]Institute of Cancer and Basic Medicine, Chinese Academy of Sciences, Cancer Hospital of the University of Chinese Academy of Sciences, Zhejiang Cancer Hospital, Hangzhou, China.
[2]College of Biomedical Engineering & Instrument Science, Zhejiang University, Hangzhou, 310027, P.R. China
[3]Schepens Eye Research Institute of Massachusetts Eye and Ear, Boston, MA. USA
[4]Department of Ophthalmology, Harvard Medical School, Boston, MA, USA
[5]School of Basic Medical Sciences, Ningxia Medical University, Yinchuan, Ningxia, P.R. China
[6]Shenzhen Eye Hospital, Shenzhen Eye Institute, Jinan University, Shenzhen, Guangdong Province, P.R. China
[7]Guangzhou Women and Children's Medical Center, Guangzhou Medical University, 510005 Guangzhou, P.R. China

***Correspondence:** 18 Zetian Road, Shenzhen Eye Hospital, Futian District, Shenzhen, Guangdong Province, P.R. China. Email address: WZ:rutin@zju.edu.cn, JW:wangjiantao@126.com, HL: leihetian18@hotmail.com

Fig. S1

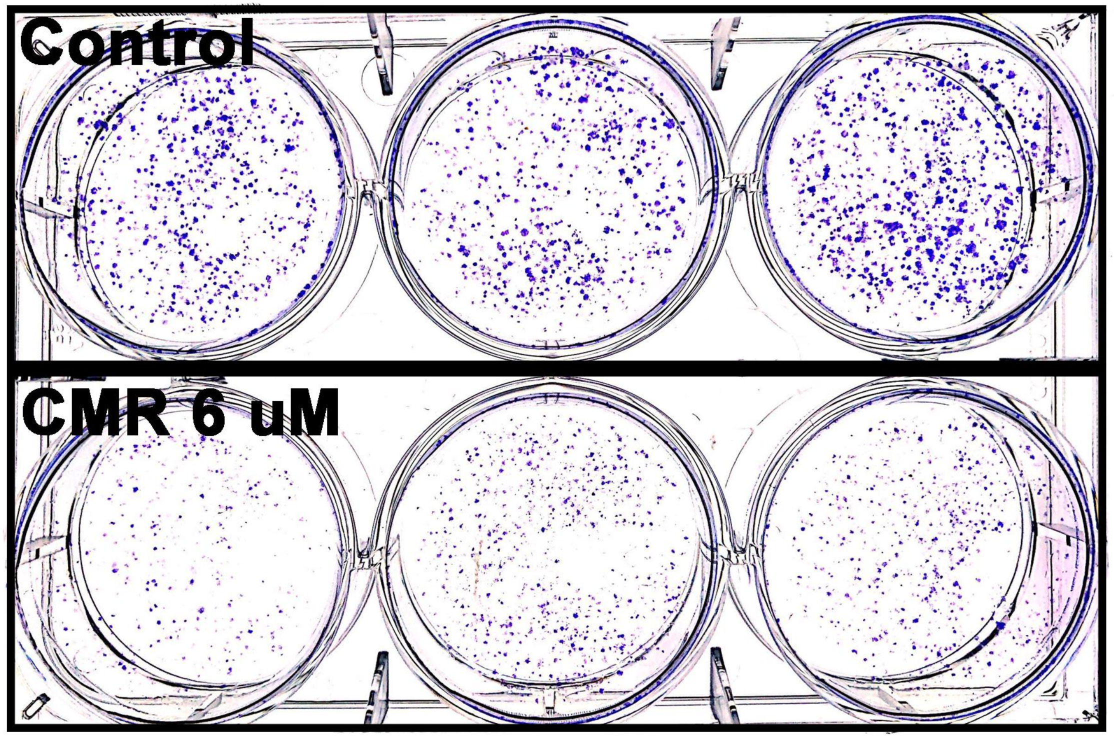

Fig. S1 CMR can inhibit proliferation of MDA-MB-231 cell.

Clonogenic assay. MDA-MB-231 cells were cultured overnight in a 6-well plate (1 $x10^4$ cells per well) and then treated with CMR 6 uM for 9 days to form colonies. Then culture medium was removed and the cell monolayer was fixed with solvent contained methanol and glacial acetic acid (3:1) for 15 min and stained with crystal violet (0.2%) for 30 min to visualize cell colonies. Each group was repeated in triplicate.

Fig. S2

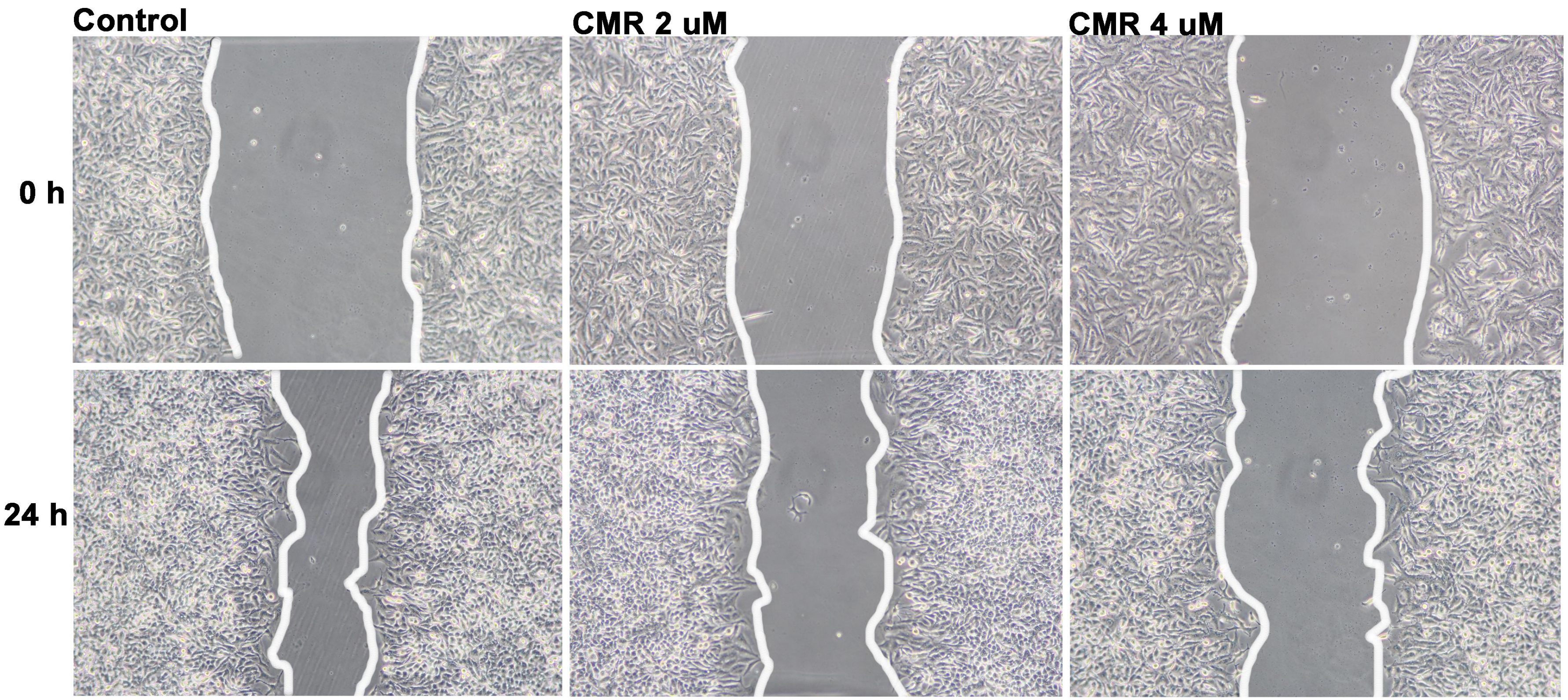

Fig. S2 CMR can inhibit migration of MDA-MB-231 cell.

When MDA-MB-231 cells were grown to confluence in 6-well plates, autoclaved 200 μl pipet tips were used to scratch the cells across the wells. The detached cells were aspirated out in PBS, and the rest cells were treated with plus or minus CMR (2 uM and 4 uM). The scratched areas were photographed to capture the initial width and photographed again 24 h later.

Fig. S3

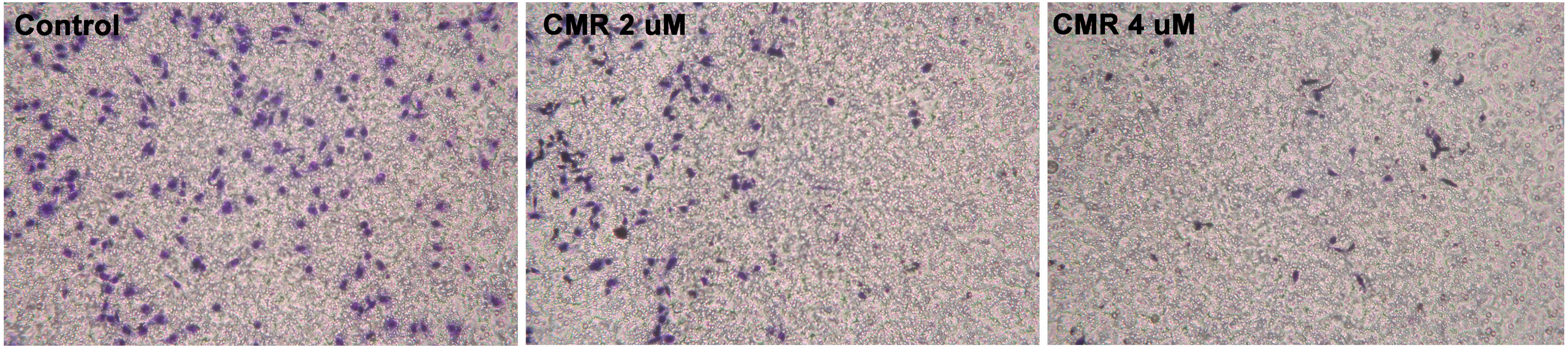

Fig. S3 CMR can inhibit invation of MDA-MB-231 cell.

Transwell assay was performed to estimate the effects of CMR on the invasion of MDA-MB-231 cells. Matrigel (5 mg/ml to 1 mg/ml) was diluted in serum free-cold DMEM medium and put 100 μl of the diluted matrigel into upper chamber of 24-well transwell. Incubated the transwell at 37 ℃ for 4 h for gelling. Cells were harvested by Trypsin/EDTA and resuspended in media containing 1% FBS at a density of 100 cells/ml. Gently wash gelled matrigel with warmed serum free media. Put 100 μl of the cell suspension onto the matrigel. 600 μl of DMEM containing 2 uM or 4 uM CMR was filled in the upper chamber. After 24 h of incubation, transwells were removed from the 24-well and cells were fixed with solvent contained methanol and glacial acetic acid (3:1) for 15 min and stained with crystal violet (0.2%) for 30 min. The noninvaded cells on the top of the transwell were scraped off using a cotton swab and the invaded cells were taken pictures under light microscope.
